# Transarterial Chemoembolization Combined with Radiofrequency Ablation in the Treatment of Stage B1 Intermediate Hepatocellular Carcinoma

**DOI:** 10.1155/2019/6298502

**Published:** 2019-09-16

**Authors:** Furong Liu, Minshan Chen, Jie Mei, Li Xu, Rongping Guo, Xiaojun Lin, Yaojun Zhang, Zhenwei Peng

**Affiliations:** ^1^Department of Medical Oncology, Sun Yat-sen University Cancer Center, State Key Laboratory of Oncology in South China, Collaborative Innovation Center for Cancer Medicine, Guangzhou 510060, China; ^2^Department of Hepatobiliary Surgery, Sun Yat-sen University Cancer Center, State Key Laboratory of Oncology in South China, Collaborative Innovation Center for Cancer Medicine, Guangzhou 510060, China; ^3^Clinical Trials Unit, The First Affiliated Hospital of Sun Yat-sen University, 58 Zhongshan Road 2, Guangzhou 510080, China; ^4^Department of Radiotherapy, The First Affiliated Hospital of Sun Yat-sen University, 58 Zhongshan Road 2, Guangzhou 510080, China

## Abstract

**Background:**

Due to the heterogeneity of patients with Barcelona clinic liver cancer (BCLC) intermediate-stage hepatocellular carcinoma (HCC), Bolondi criteria were proposed and patients were divided into four substages. The purpose of this study was to compare the survival of substage B1 patients who were initially treated with a combination of transarterial chemoembolization (TACE) and radiofrequency ablation (RFA) (TACE-RFA) or TACE alone.

**Methods:**

404 patients with stage B1 HCC were retrospectively analyzed from January 2005 to December 2012. 209 patients received TACE-RFA, and 195 received TACE alone as initial treatment. The overall survival (OS) and progression-free survival (PFS) rates were estimated by the Kaplan–Meier method and compared by the log-rank test.

**Results:**

1-, 3-, and 5-year OS rates were 83.7%, 45.8%, and 24.8% in the TACE-RFA group and 80.7%, 26.4%, and 16.7% in the TACE group, respectively (*P*=0.003). The corresponding PFS rates were 71.8%, 26.6%, and 13.0% and 59.1%, 11.0%, and 2.2% in the TACE-RFA group and TACE group, respectively (*P* < 0.001). Multivariate regression analysis indicated that tumor size (OS: hazard ratio (HR) = 0.683, *P*=0.001; PFS: HR = 0.761, *P*=0.013), along with treatment allocation (OS: HR = 0.701, *P*=0.003; PFS: HR = 0.620, *P* < 0.001), was the independent prognostic factor for both OS and PFS.

**Conclusions:**

Combination TACE and RFA treatment yielded better survival than TACE alone for patients with stage B1 HCC according to the Bolondi criteria.

## 1. Introduction

Hepatocellular carcinoma (HCC) is the sixth most common cancer and the third leading cause of cancer-related death worldwide [1], and the Barcelona clinic liver cancer (BCLC) system is one of the most widely and frequently used staging system of HCC [2].

Transarterial chemoembolization (TACE) is the recommended treatment for HCC patients with the intermediate stage (BCLC stage B) according to the BCLC staging system, which constitutes ∼30% of all stages of HCC [3–6]. However, patients with BCLC stage B HCC are highly heterogeneous for they present differences in tumor burden, liver function, and possible comorbidities [2, 6]. Therefore, a single treatment is hard to address all the needs. Other treatments such as liver resection and transplantation had showed superior outcomes to TACE when applied to highly selected groups [7–10]. Considering these available options, the BCLC strategy of stage B HCC needs further refinement. The up-to-7 criteria are emerging as a tumor burden assessment method which require the sum of the number of tumors and the largest tumor size being no more than 7 [11]. The stage B HCC can be divided into four categories (stages B1 to B4 HCC) based on “up-to-7 criteria,” liver function evaluated by the Child–Pugh score and patients' performance status (PS) scored by ECOG (Eastern Cooperative Oncology Group) according to the Bolondi criteria [12–14]. For patients with stage B1 HCC, who are with a PS score of 0-1, a Child–Pugh score of 5–7, and tumors within the up-to-7 criteria, TACE is still a dominant treatment [12]. However, TACE alone is usually hard to make the target lesion necrose completely, so repeated procedures are performed to achieve maximum tumor recession, but then liver function is progressively deteriorated [15, 16]. Therefore, combining TACE with other effective treatments with an intention to cure is becoming an alternative option for patients with intermediate-stage HCC [12]. Radiofrequency ablation (RFA) is a curative treatment for very early- and early-stage HCC [2–4]. Combination of TACE and RFA (TACE-RFA) treatment has achieved comparable survivals in liver resection for HCC within and even beyond the Milan criteria [17–19]. It also demonstrated better control of tumor less than 7 cm compared with RFA alone [20], making it a potential curative strategy for selected HCC [21]. However, whether TACE-RFA treatment could gain therapeutic benefits for stage B1 HCC patients has not been fully clarified.

The purpose of the study was to evaluate the long-term survival of TACE-RFA treatment in the treatment of patients with stage B1 HCC.

## 2. Materials and Methods

### 2.1. Patients

This study was a retrospectively analysis of data collected from our prospective database from January 2005 to December 2012. Approval was obtained from the institution review board, and informed consent was waived. 404 patients received either TACE-RFA treatment or TACE treatment as first-time treatment and were enrolled according to the following eligibility criteria: (1) age 18–75 years, (2) HCC diagnosed by pathology or dynamic computed tomography (CT) or magnetic resonance imaging (MRI) showing typical features [22], (3) no previous antitumor treatment, (4) tumor burden beyond the Milan criteria and within up-to-7 criteria [12], (5) and a Child–Pugh score of 5–7. And the exclusion criteria were as follows: (1) presence of simultaneous carcinoma, (2) ECOG score ≥1, (3) existing severe coagulation disorders such as prothrombin activity <40% or platelet count <40,000/*μ*L; (4) existing hepatic decompensation such as ascites refractory to diuretics.

### 2.2. Definition of Stage B1 HCC

Tumor burden of B1 HCC was assessed using both the Milan criteria and the up-to-7 criteria. The threshold of the Milan criteria is one tumor smaller than 5 cm or up to 3 tumors each smaller than 3 cm, whereas the up-to-7 criteria add the number of tumors to the size of the largest one by centimeter, with the sum being less than 7. Patients with a Child–Pugh score of 5–7, ECOG score 0, and tumor burden beyond the Milan criteria and within up-to-7 criteria were classified as stage B1 HCC [12, 13].

### 2.3. Treatments

Before these patients underwent initial treatment, our HCC multidisciplinary treatment team, which included hepatobiliary surgeons, medical oncologists, interventional radiologists, and diagnostic radiologists, discussed treatment for each patient. Patients were recommended to choose a treatment strategy of either TACE-RFA or TACE alone and be noted by the efficacy, comorbidity, and cost of both treatments. If the patients chose TACE-RFA, RFA was performed within 1-2 weeks after TACE. The timing of RFA after TACE was based on the disappearance of postembolization syndrome and the recovery of liver dysfunction. Patients who refused RFA underwent TACE only.

#### 2.3.1. TACE Procedure

TACE was performed as previously described by our team [20, 23]. In brief, after introduction of a 5-F catheter into the celiac trunk using the Seldinger technique through the femoral artery, an angiography was performed to confirm the patency of the portal vein and assess the location, size, and artery supply of the tumors, and then the catheter was advanced to the tumor-feeding segmental arteries for embolization of all tumors. Carboplatin (300 mg) was infused first, and then a mixture of epirubicin (50 mg), mitomycin (8 mg), and lipiodol (5 ml) was infused. The amounts of chemotherapy agents and oil were identical for all cases. Absorbable gelatin sponge particles (1 to 2 mm in diameter) were used for final embolization. An angiography survey was performed to assess the extent of vascular occlusion and blood flow in other arterial vessels.

#### 2.3.2. RFA Procedure

A dynamic CT or MRI scan was performed to preliminary assess whether the tumor response was progression disease and the presence of significant complications, such as ectopic embolism, liver ischemia, and bleeding after TACE treatment. RFA was performed within 2 weeks (median, 8 days; range, 7–14 days) after TACE under real-time ultrasound guidance and general anesthesia by using an alternating current generator (RF3000; Boston Scientific, Boston, MA, USA) and an electrode needle with an insulated 15-gauge outer needles which houses 10 solid retractable curved electrodes, with a diameter of 3.5 cm when expanded like an umbrella. The ablation system is based on tumor impedance. A marked increase of impedance is considered as successfully ablated; if not, a second application of ablation would be given. Multiple overlapping ablation was performed for tumors with the greatest dimension of 3.0 cm, which was described by Chen et al. [24].

### 2.4. Complications and Follow-up

Complications were evaluated by using the National Cancer Institute Common Toxicity Criteria grading version 4.0 [25] by two authors. Any disagreement was settled by counselling a third author.

A contrast-enhanced abdominal CT or MRI scan was done 4 weeks after TACE-RFA or TACE treatment and thereafter once every 3-4 month for the first 2-year. Routine tests including liver function and serum alpha fetoprotein (AFP) were examined at each follow-up visit. Chest X-ray was done once every 6 months. For patients with a suggestion of extrahepatic metastasis, a CT of the chest and/or bone scintigraphy would be performed. Follow-up intervals were 6 months during 2–5 years after treatment and then 12 months after 5 years. The last follow-up time was December 31, 2017.

Overall survival (OS) was calculated from the diagnostic date to the date of death or the last follow-up. Progression-free survival (PFS) was calculated from the initial treatment date to radiologic disease progression according to modified response evaluation criteria in solid tumors (mRECISTs) [26], the date of death, or the date of last follow-up. If tumor progression was present in either group during follow-up, subsequent treatments will be applied.

### 2.5. Statistical Analysis

Comparisons were made by using Student's *t* test for continuous variables and the chi-square test or Fisher's exact test for categorical variables as appropriate. Survivals were calculated by the Kaplan–Meier survival method and compared by the log-rank test. Prognostic significance factors were analyzed by a Cox proportional hazard regression model. Statistical significance was set at a two-tailed *P* < 0.05. All statistical analyses of the data were performed with SPSS 20.0 statistical software (SPSS, Inc., Chicago, IL, USA).

## 3. Results

### 3.1. Patient Characteristics

Patients' baseline characteristics are summarized in Table 1. A total of 404 patients with stage B1 HCC were included in this study. Patients were predominantly male in the TACE-RFA group (*n* = 184, 88.0%) and the TACE group (*n* = 165, 84.6%). The median age in the TACE-RFA and the TACE group was 52.9 and 58.7 years, respectively. The dominant etiology of liver disease was hepatitis B virus infection in both groups, with 180 (86.1%) patients in the TACE-RFA group and 176 (90.3%) in the TACE group. In addition, 108 (51.7%) patients received antiviral treatment for hepatitis B in the TACE-RFA group and 99 (50.8%) did in the TACE group. There was no significant difference in alanine transaminase (ALT), albumin, total bilirubin, *γ*-glutamyl transpeptidase, platelet count, prothrombin activity, AFP, Child–Pugh class, tumor size, and tumor number between the two groups.

### 3.2. Complications

No treatment-related deaths occurred in both groups. Common complications including fever, pain, ascites, vomiting, and pleural effusion were observed in both groups. Severe complications were occurred in both groups including gastric hemorrhage, bile duct stenosis, abdominal infection, and small intestinal obstruction. No significant difference was showed in the types and grades of the complications between the two groups (Table 2).

### 3.3. Survivals

**Figure 1:**
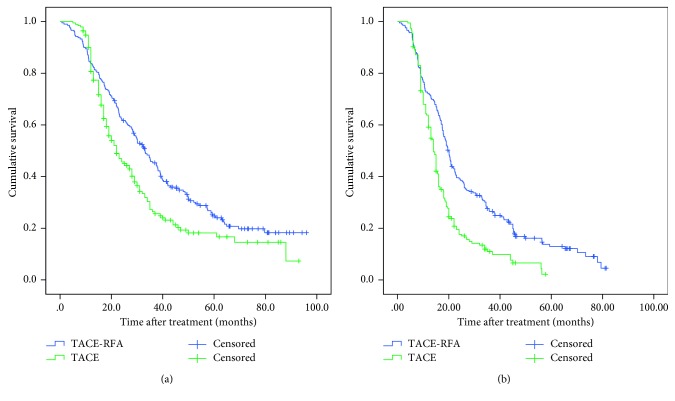
Kaplan–Meier curves of (a) overall survival and (b) progression-free survival after TACE-RFA and TACE.

156 (74.6%) patients in the TACE-RFA group and 133 (68.2%) patients in the TACE group died during follow-up. The causes of death are shown in [Supplementary-material supplementary-material-1]. The median OS time was 33.1 ± 2.5 (95% CI: 28.1–38.0) months and 22.0 ± 2.3 (95% CI: 17.4–26.6) months in the TACE-RFA group and the TACE group, respectively. The 1-, 3-, and 5-year OS rates were 83.7%, 45.8%, and 24.8% in the TACE-RFA group, while the corresponding OS rates were 80.7%, 26.4%, and 16.7% in the TACE group, respectively (Figure 1(a), *P*=0.003).

109 (52.2%) patients in the TACE-RFA group and 118 (60.5%) in the TACE group (*P*=0.108) presented tumor progression during the follow-up. The most prevalent type of tumor progression was intrahepatic progression. The details of types and treatments of tumor progression are listed in Table 3. More patients in the TACE-RFA group received RFA for tumor progression after initial treatment than in the TACE group (*P*=0.031).

The median PFS of the TACE-RFA group and TACE group was 20.0 ± 0.8 (95% CI: 18.5–21.5) months and 14.0 ± 0.6 (95% CI: 15.2–17.8) months, respectively. The cumulative 1-, 3-, and 5-year PFS rates were 71.8%, 26.6%, and 13.0% in the TACE-RFA group and 59.1%, 11.0%, and 2.2% in the TACE group, respectively (Figure 1(b), *P* < 0.001).

The results of subgroup analysis of survivals are presented in Table 4. For patients with tumor ≤3 cm or solitary tumor, the TACE-RFA group had better OS than TACE alone (*P*=0.024 and *P*=0.016, respectively). The TACE-RFA group yielded better PFS in all subgroups than TACE alone.

### 3.4. Multivariate Analysis

By univariate analysis, total bilirubin (*P*=0.022), prothrombin activity (*P*=0.027), serum ALT (*P*=0.018), tumor size (*P*=0.001), and treatment allocation (*P*=0.003) showed relevance to OS; meanwhile, serum AFP (*P*=0.041), number of tumors (*P*=0.024), tumor size (*P*=0.009), and treatment allocation (*P* < 0.001) showed relevance to PFS. Multivariate analysis with the Cox proportional hazard model indicated that tumor size (OS: HR = 0.683, 95% CI, 0.541–0.862, *P*=0.001; PFS: HR = 0.761, 95% CI, 0.613–0.944, *P*=0.013), along with treatment allocation (OS: HR = 0.701, 95% CI, 0.554–0.888, *P*=0.003; PFS: HR = 0.620, 95% CI, 0.487–0.789, *P* < 0.001), was the independent prognostic factors for both OS and PFS (Table 5).

## 4. Discussion

Considering the marked heterogeneity of BCLC B stage HCC patients, Bolondi et al. divided BCLC B HCC patients into four subgroups and suggested possible treatment for each substage to facilitate clinical decisions [12]. TACE was the recommend treatment for substage B1 HCC. However, whether combined TACE with RFA could prolong survival for patients with substage B1 HCC was unclear. Our study indicated that combination of TACE and RFA treatment achieved better OS and PFS than TACE alone for patients with substage B1 HCC, and multivariate analysis revealed that tumor size and treatment allocation were significant prognostic factors for both OS and PFS.

Combined TACE and RFA has become a common treatment for HCC especially for medium-sized and multinodule tumors [18–20]. Pan et al. [18] found that TACE-RFA provided comparable OS rates than that of surgical resection in patients within the up-to-7 criteria. Besides, another study showed that TACE-RFA can prolong OS of patients with BCLC B HCC compared with TACE alone [19]. Our results also found TACE-RFA was a more effective treatment compared with TACE, but we performed the combination treatment in subgroup HCC patients based on the Bolondi criteria which not only took tumor load in consideration but also liver function and patients' performance status. However, the 5-year OS and PFS rates of the TACE-RFA group in our study were 24.8% and 13.0%, which were lower than that of patients in Pan's study (5-year OS: 41.3%; 5-year PFS 20.8%) [18]. The main reason could be that our study had more patients presenting multitumor (72.2% vs. 31.1%), tumor load beyond the Milan criteria (100% vs. 36.9%), and liver function at Child–Pugh B (9.6% vs. 2.9%) in the TACE-RFA group, compared with Pan's study. In our study, 52.2% of patients in the TACE-RFA group and 60.5% in the TACE group suffered tumor progression, and intrahepatic recurrence was the dominant pattern of tumor progression. As for treatment of tumor progression, patients in the TACE-RFA group received more RFA treatment than patients in the TACE group (*P*=0.031). The results showed that TACE-RFA treatment could achieve better tumor control, even provided the chance to receive additional curative treatment when tumor progressed.

We then analyzed the influence of tumor size and number on long-time survival. Indeed, we found that, for patients with solitary tumor and patients with tumor ≤3 cm, TACE-RFA treatment showed better OS than TACE treatment. In our study, patients with single tumor indicated that the tumor size could be less than 6 cm but bigger than 5 cm. An RCT revealed that TACE-RFA had a preferable survival in treating patients with single HCC tumor size up to 7 cm [20], which was consistent with our results. As for patients with tumor ≤3 cm, the tumor number was four, and combination treatment had better survivals maybe because RFA can be applied on all lesions and was not inferior to resection of HCC tumor ≤3 cm [3, 4]; in addition, TACE can play therapeutic treatment and enhance the effect of RFA. But the TACE-RFA group did not disclose the same outcomes of OS for patients with tumor >3 cm and patients with more than one tumor with the *P* value being borderline significant. The subgroup analysis of RFS showed that TACE-RFA treatment achieved better RFS in either tumor size and tumor number. The synergy effects of TACE derived ischemic cytotoxic- and RFA-induced thermal damage which can be applied to explain the following results: (1) TACE can reduce the cooling effect that subsequent RFA confronted, (2) the ischemia and inflammation that were produced by TACE are expected to enlarge the area of necrosis and therefore increase the safety margin [27], and (3) heat deposition in the treatment area by the RFA procedure enhanced the efficiency of chemotherapeutics used in TACE [28].

Although patients in the TACE-RFA group received two different local procedures, the incidence of treatment-related complications were equivalent to those in the TACE group. TACE-RFA treatment was safe, and the results were consistent with other studies [17–20].

Our study has some limitations. First, selection bias existed due to the retrospective nature of the study. The treatment options either TACE-RFA or TACE alone in HCC B1 stage patients were individually determined based on the discussion of the HCC multidisciplinary treatment team or the patient; this mostly resulted in a selection bias in the study. Furthermore, this study was conducted only in our institution, and the results of the study could be influenced by their own experience. Second, the response of TACE was not in analysis in our study. Although a dynamic CT or MRI scan was done after TACE within 7 days, the purpose was to assess patients with progression disease and significant complications of TACE. Whether the patients with better response of TACE gained better survivals in the TACE-RFA group than TACE alone or not was unclear, and future study needs to be performed to verify. Third, the etiology of HCC disease was mostly chronic hepatitis B infection, and the results needed to be further validated in patients with hepatitis C infection, alcoholic liver disease, fatty liver disease, and cryptogenic disease.

In conclusion, the combination of TACE and RFA treatment achieved better survival than TACE alone for patients with stage B1 HCC, especially for patients with tumor ≤3 cm and patients with solitary tumor.

## Figures and Tables

**Table 1:** Baseline characteristics for all patients.

Variables	TACE-RFA (*n* = 209)	TACE (*n* = 195)	*P* value
Gender (male/female)	184/25	165/30	0.384
Age (years)^#^	59.2 ± 4.0 (18–75)	58.7 ± 4.0 (20–75)	0.872
HBsAg (+/−)	180/29	176/19	0.221
HCV (+/−)	10/199	7/188	0.625
ALT (U/L)^#^	27.2 ± 12.0 (16.5–78.2)	29.0 ± 11.9 (15.9–77.0)	0.789
ALB (g/L)^#^	35.7 ± 3.0 (33.0–49.0)	35.9 ± 3.4 (32.4–47.0)	0.876
Total bilirubin (*μ*mol/L)^#^	12.8 ± 4.2 (7.0–22.0)	12.3 ± 4.5 (6.3–22.0)	0.902
GGT (U/L)^#^	106.0 ± 51.0 (49.0–422.0)	103.5 ± 61.0 (52.5–434.3)	0.675
Platelet count (×10^9^/L)^#^	125.2 ± 25.9 (75–401)	130.2 ± 28.2 (80–403)	0.710
Prothrombin activity (%)^#^	98.0 ± 19.0 (78–120)	98.0 ± 18.4 (77–119)	0.735
AFP (ng/ml)			0.762
≤400	123	111	
>400	86	84	
Tumor size (cm)			0.840
≤3	125	114	
>3	84	81	
Tumor number			0.447
1	58	61	
>1	151	134	
Child–Pugh class (A/B)	189/20	180/15	0.596
Antiviral treatment for hepatitis B (yes/no)	108/101	99/96	0.921

^#^Data are represented in mean ± standard deviation. Data in parentheses are ranges. Except where indicated, data values represent the number of patients. TACE: transarterial chemoembolization; RFA: radiofrequency ablation; HBsAg: hepatitis B surface antigen; HCV: hepatitis C virus; ALT: alanine aminotransferase; ALB: albumin; GGT: *γ*-glutamyl transpeptidase; AFP: alpha fetoprotein.

**Table 2:** Treatment-related adverse events.

	TACE-RFA (*n* = 209)	TACE (*n* = 195)	*P* value
	Grade 1-2/3-4	Grade 1-2/3-4	
Pain	120/4	103/2	0.690
Fever (temperature >38.5°C)	76/1	59/1	0.999
Vomiting	86/10	70/7	0.804
Ascites	6/1	6/1	0.999
Pleural effusion	12/1	15/2	0.999
Bile duct stenosis	2/0	1/0	0.999
Gastric hemorrhage	2/0	1/1	0.999
Abdominal infection	0/1	0/1	0.999
Small intestinal obstruction	0/1	0/0	0.999

Data values represent the number of patients. TACE: transarterial chemoembolization; RFA: radiofrequency ablation.

**Table 3:** Types and treatments of tumor progression.

	TACE-RFA (*n* = 209)	TACE (*n* = 195)	*P* value
*Types of tumor progression*			
Intrahepatic	92	94	0.608
Extrahepatic	14	18	0.385
Both	3	6	0.327

*Treatment of tumor progression*			
RFA	21	8	0.031
TACE	64	85	0.067
Sorafenib	19	17	1.000
Chemotherapy	4	5	0.745
Conservative treatment	1	3	0.359

Data values represent the number of patients. TACE: transarterial chemoembolization; RFA: radiofrequency ablation.

**Table 4:** Subgroup analysis of survivals.

	TACE-RFA				TACE				*P* value
	1-year (%)	3-year (%)	5-year (%)	Median time (months)	1-year (%)	3-year (%)	5-year (%)	Median time (months)	
*Overall survival*									
Tumor size ≤3 cm	88.8	51.3	30.8	37.6	80.5	31.8	23.8	26.0	0.024
Tumor size >3 cm	75.0	37.9	14.3	29.8	75.5,	18.8	11.0	19.0	0.053
Single tumor	86.2	53.9	28.4	37.8	75.2	27.1	17.6	23.0	0.016
Multiple tumors	82.8	42.7	23.4	29.8	73.5	26.0	13.6	22.0	0.051

*Progression-free survival*									
Tumor size ≤3 cm	75.2	32.1	15.3	21.9	66.6	14.9	3.1	15.0	<0.001
Tumor size >3 cm	66.7	18.4	9.9	18.0	54.8	8.7	0	13.0	0.008
Single tumor	79.3	31.9	17.2	21.0	59.7	14.3	4.8	14.2	0.001
Multiple tumors	69.5	24.5	11.6	19.0	55.9	9.4	0	13.8	<0.001

TACE: transarterial chemoembolization; RFA: radiofrequency ablation.

**Table 5:** Univariate and multivariate analyses of predictors of overall survival and progression-free survival after treatment.

Factors	Overall survival				Progression-free survival			
	Univariate	Multivariate			Univariate	Multivariate		
	*P* value	HR	95% CI	*P* value	*P* value	HR	95% CI	*P* value
Gender (male/female)	0.897				0.509			
Age (years)	0.690				0.839			
HBsAg (+/−)	0.654				0.543			
HCV (+/−)	0.876				0.737			
Platelet count (×10^9^/L) (≤100/>100)	0.285				0.187			
Child–Pugh class (A/B)	0.688				0.527			
Total bilirubin level (*μ*mol/L) (≤17.1/>17.1)	0.022				0.109			
ALB level (g/L) (≤35/>35)	0.678				0.729			
Prothrombin activity (%) (≤100/>100)	0.027				0.067			
ALT (IU/L) (≤40/>40)	0.018				0.178			
AFP (ng/mL) (≤400/>400)	0.286				0.041			
Number of tumors (1/>1)	0.326				0.024			
Tumor size (≤3/>3 cm)	0.001	0.683	0.541–0.862	0.001	0.009	0.761	0.613–0.944	0.013
Treatment allocation (TACE-RFA/TACE)	0.003	0.701	0.554–0.888	0.003	<0.001	0.620	0.487–0.789	<0.001
Antiviral treatment for hepatitis B (yes/no)	0.967				0.854			

TACE: transarterial chemoembolization; RFA: radiofrequency ablation; HR: hazard ratio; CI: confidence interval; HBsAg: hepatitis B surface antigen; HCV: hepatitis C virus; ALT: alanine aminotransferase; ALB: albumin; GGT: *γ*-glutamyl transpeptidase; AFP: alpha fetoprotein.

## Data Availability

The clinical data used to support the findings of this study were provided by Department of Hepatobiliary Surgery, Sun Yat-sen University Cancer Center, and cannot be made freely available. Access to these data will be considered by the corresponding author upon request, with permission of director of Department of Hepatobiliary Surgery.

## Supplementary Materials

Supplementary Materials Table S1: cause of death during follow-up.
