# Supplementary material for: Transarterial Chemoembolization Combined with Radiofrequency Ablation in the Treatment of Stage B1 Intermediate Hepatocellular Carcinoma
Source: J Oncol. 2019 Sep 16;2019:6298502. doi: 10.1155/2019/6298502 (PMC6766138; doi:10.1155/2019/6298502)
Supplement: Supplementary Materials — Table S1: cause of death during follow-up. [file 6298502.f1.docx]

**Table S1. Cause of death during follow-up**

| Cause of death | TACE-RFA (n=209) | TACE (n= 195) | *P* value |
| --- | --- | --- | --- |
| Tumor progression | 109 | 118 | 0.407 |
| Liver failure with stable tumor | 41 | 10 | 0.037 |
| Variceal bleeding | 5 | 3 | 0.999 |
| Others | 1 | 2 | 0.275 |
